# The therapeutic potential of the novel angiotensin-converting enzyme 2 in the treatment of coronavirus disease-19

**DOI:** 10.14202/vetworld.2021.2705-2713

**Published:** 2021-10-23

**Authors:** Ademola Adetokunbo Oyagbemi, Temitayo Olabisi Ajibade, Yapo Guillaume Aboua, Idayat Titilayo Gbadamosi, Aduragbenro Deborah A. Adedapo, Abimbola Obemisola Aro, Olumuyiwa Abiola Adejumobi, Emma Thamahane-Katengua, Temidayo Olutayo Omobowale, Olufunke Olubunmi Falayi, Taiwo Olaide Oyagbemi, Blessing Seun Ogunpolu, Fasilat Oluwakemi Hassan, Iyanuoluwa Omolola Ogunmiluyi, Olufunke Eunice Ola-Davies, Adebowale Benard Saba, Adeolu Alex Adedapo, Sanah Malomile Nkadimeng, Lyndy Joy McGaw, Prudence Ngalula Kayoka-Kabongo, Momoh Audu Yakubu, Oluwafemi Omoniyi Oguntibeju

**Affiliations:** 1 Department of Veterinary Physiology and Biochemistry, Faculty of Veterinary Medicine, University of Ibadan, Nigeria; 2 Department of Health Sciences, Faculty of Health and Applied Sciences, Namibia University of Science and Technology, Private Bag 13388, Namibia; 3 Department of Botany, Faculty of Science, University of Ibadan, Nigeria; 4 Department of Pharmacology and Therapeutics, University of Ibadan, Nigeria; 5 Department of Agriculture and Animal Health, College of Agriculture and Environmental Sciences, University of South Africa, Florida, South Africa; 6 Department of Veterinary Medicine, Faculty of Veterinary Medicine, University of Ibadan, Nigeria; 7 Department of Health Information Management, Botho University, Faculty of Health and Education, Botswana; 8 Department of Veterinary, Faculty of Veterinary Medicine, University of Ibadan, Nigeria; 9 Department of Veterinary Pharmacology and Toxicology, Faculty of Veterinary Medicine, University of Ibadan, Nigeria; 10 Department of Veterinary Parasitology and Entomology, Faculty of Veterinary Medicine, University of Ibadan, Nigeria; 11 Department of Paraclinical Science, Phytomedicine Programme, University of Pretoria, Faculty of Veterinary Science, Old Soutpan Road, Onderstepoort, 0110, South Africa; 12 Department of Environmental and Interdisciplinary Sciences, College of Science, Engineering and Technology, Vascular Biology Unit, Center for Cardiovascular Diseases, Texas Southern University, Houston, TX, USA; 13 Department of Biomedical Sciences, Phytomedicine and Phytochemistry Group, Oxidative Stress Research Centre, Faculty of Health and Wellness Sciences, Cape Peninsula University of Technology, Bellville 7535, South Africa.

**Keywords:** Renin-angiotensin system, COVID-19, hypertension, lung injury, ACE2, SARS-CoV-2

## Abstract

Severe acute respiratory syndrome coronavirus 2 (SARS-CoV-2) is the etiological agent of coronavirus disease 2019 (COVID-19). This virus has become a global pandemic with unprecedented mortality and morbidity along with attendant financial and economic crises. Furthermore, COVID-19 can easily be transmitted regardless of religion, race, sex, or status. Globally, high hospitalization rates of COVID-19 patients have been reported, and billions of dollars have been spent to contain the pandemic. Angiotensin-converting enzyme (ACE) 2 is a receptor of SARS-CoV-2, which has a significant role in the entry of the virus into the host cell. ACE2 is highly expressed in the type II alveolar cells of the lungs, upper esophagus, stratified epithelial cells, and other tissues in the body. The diminished expressions of ACE2 have been associated with hypertension, arteriosclerosis, heart failure, chronic kidney disease, and immune system dysregulation. Overall, the potential drug candidates that could serve as ACE2 activators or enhance the expression of ACE2 in a disease state, such as COVID-19, hold considerable promise in mitigating the COVID-19 pandemic. This study reviews the therapeutic potential and pharmacological benefits of the novel ACE2 in the management of COVID-19 using search engines, such as Google, Scopus, PubMed, and PubMed Central.

## Introduction

Severe acute respiratory syndrome coronavirus (SARS-CoV-2) is the etiological agent for coronavirus disease 2019 (COVID-19). The virus was first detected in Wuhan, China, in December 2019 and was later declared by the WHO as a global pandemic in March 11, 2020. SARS-CoV-2 is a highly infectious virus with an extremely high degree of transmissibility. Globally, as of June 20, 2021, 177,108,695 cases of COVID-19 have been confirmed, including 3,840,223 deaths. Currently, in Nigeria, more than 167,206 cases with over 2117 deaths due to COVID-19 were reported. However, a total number of 163,550 patients have recovered.

In the past few weeks, significant achievements have been recorded in vaccine production technology. Fortunately, vaccines from Pfizer and BioNTech (USA/Germany), Moderna (USA), Johnson and Johnson (USA), AstraZeneca, (UK), Sinovac (China), and Sputnik V (Russia) have received emergency authorization for use in the UK, the USA, France, China, Africa, and other countries in the world.

However, the emergence of a new variant of SARS-CoV-2 has put the world in an unprecedented vicarious state. As of June 20, 2021, more than 2.59 billion vaccine doses have been administered worldwide. Therefore, this review aims to investigate the therapeutic potential of the novel angiotensin-converting enzyme 2 (ACE2) in the fight against the global COVID-19 pandemic.

## Renin–angiotensin System (RAS) and Novel ACE2

**Figure-1:**
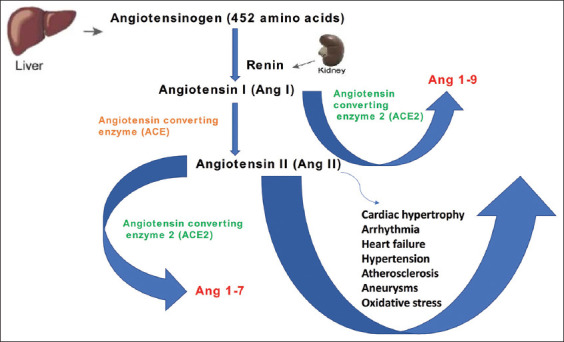
The components of renin-angiotensin system (RAS) and its involvement in the cardiovascular system.

**Figure-2:**
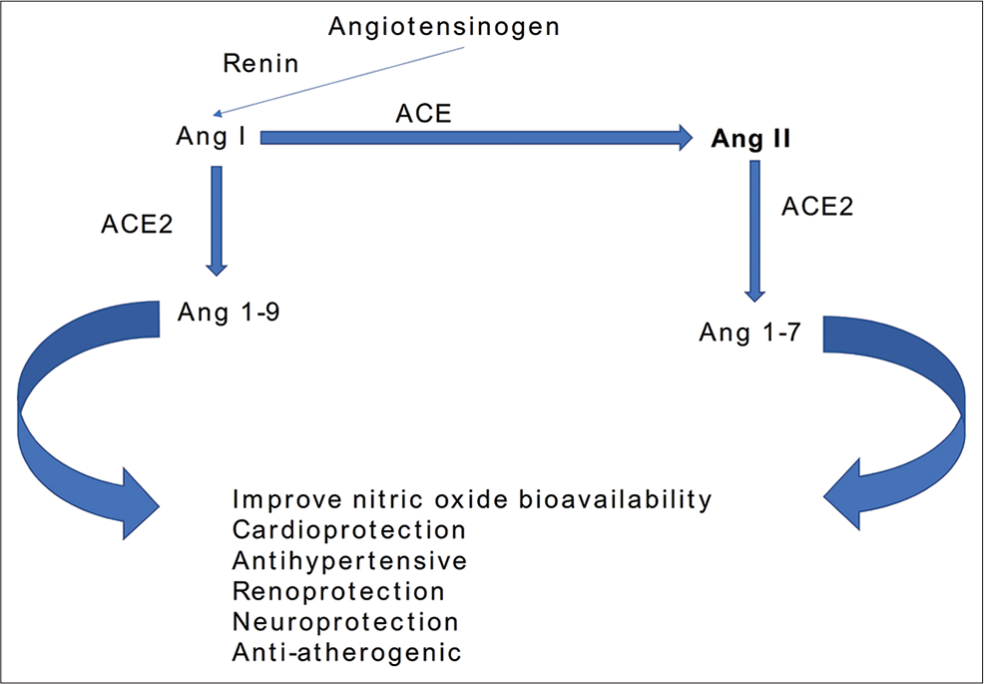
Catabolism of angiotensinogen and the catabolic products of novel angiotensin-converting enzyme 2.

Angiotensinogen (AGT) is a hepatic a-2-globulin cleaved by renal renin to produce angiotensin (Ang) I, a precursor of the active peptide of the RAS (Figures-1 and 2). Unlike AGT which has 452 amino acids, angiotensin I is a decapeptide. The proteolytic action of the ACE produces angiotensin II from its precursor molecule angiotensin I. Ang II mediates its action through the modulation of angiotensin 1 (AT1R) and (AT2R) 2 receptors, respectively (Figure-1). AGT, along with other biological molecules of the renin–angiotensin–aldosterone system, tightly regulates the blood volume and pressure in the mammalian cardiovascular system by altering the physiological activities of the kidneys, lungs, blood vessels, and brain [1,2]. AT1 has two functional receptors, AT1a and AT1b, which are structurally similar but functionally different in their distribution in various tissues and organs [3,4]. In contrast to the AT2 receptor that is only present *in utero*, the AT1a receptor is believed to mediate several physiological and pharmacological effects postnatally [5] (Figure-2).

The potential therapeutic usefulness of ­zinc-dependent metalloproteinase ACE2 in the management of cardiovascular diseases and SARS has been well documented [6,7]. ACE2 is also an important modulator of the intestinal and renal absorption of protein. ACE2 is a glycoprotein with extracellular (catalytic metallopeptidase) domain and an intracellular carboxyl terminus tail. The extracellular portion shares a structural similarity with collectrin, which is responsible for the carboxypeptidase activity of ACE2. The controlling effect of the RAS under physiological condition is primarily associated with the activation of ACE and formation of angiotensin II, with the consequent induction of generalized vascular constriction throughout the cardiovascular system [8,9]. As a result, blood pressure is prevented from falling below the physiological range for adequate tissue perfusion. Therefore, the activity of this system is negatively regulated by ACE2, with the subsequent homeostasis of angiotensin II [10].

Chromosome Xp22 has been shown to house the ACE2 gene, which encodes the ACE2 protein comprising 805 amino acids in humans [11]. The activation of RAS mainly by the derangement of the normal cardiovascular function, such as hypovolemia and/or generalized vasodilation, leads to the production of angiotensin II from AGT in a chain reaction involving renin and ACE [12]. On the other hand, the heptapeptide Ang III with similar biological activity was discovered following the removal of aminoterminal aspartate from Ang II [12]. Similarly, Ang 1-7 were formed by the removal of amino acid phenylalanine from Ang II [13,14]. In contrast to ACE, which removes a dipeptide from the C termini of substrates, ACE2 removes one amino acid, thereby degrading Ang II with the consequent formation of the nonapeptide Ang 1-9 (Figures-1 and 2). Considering these observations, ACE2 was found to convert the Ang II actions to those of Ang 1-7, including vasodilatory, antifibrotic, anti-inflammatory, and cardioprotective effects [15,16]. The alterations in the ACE2 level have been implicated in the pathogenesis and organ-specific pathological observations of the cardiac, renal, and respiratory tissues, as exemplified by the current COVID-19 pandemic [17]. Recently, a prominent pathological role was reported for ACE2 in the transmission of SARS coronavirus, wherein ACE2 not only acts as a receptor but is also important for viral entry [18].

## ACE2 and MAS1 Proto-oncogene

The Mas receptor was first reported as a proto-oncogene that is highly expressed in the renal, adrenal, and vascular tissues [19]. However, Ang 1-7, the physiological ligand for Mas receptor, act as a vasodepressor through the reduction of peripheral resistance, thus inducing a clinically significant reduction in the blood pressure parameters [20]. A positive modulatory role of ACE 2 and Ang 1-7 in fetal development has been reported, with the reduced expression of Ang 1-7 being associated with higher cardiometabolic risk and the precipitation of non-cancerous prostatic hyperplasia in adulthood [21]. Furthermore, the non-canonical pathway of ACE2, Mas, and Ang 1-7 is known to increase the vessel diameter in the presence of cellular proliferation and growth inhibition while exerting cardiorenal protective activity [22,23].

**Figure-3:**
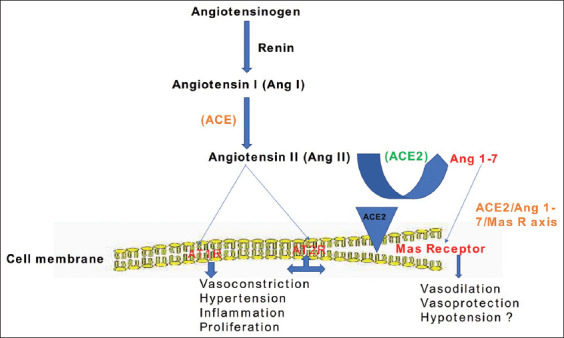
The potential benefits of angiotensin-converting enzyme 2/angiotensin-(1-7)/Mas axis and the implications of angiotensin type 1 receptor (AT1R).

Likewise, Prestes *et al*. [24] suggested the positive modulation of fibrotic tissue development by ACE2 and Mas *in vitro* (Figure-3), while Ang 1-7 have been implicated in several diseases associated with cardiac and vascular derangements. Durand *et al*. [25] reported the opposing effect of Ang 1-7 as a vasodilator mediated by Mas and Ang II (potent vasoconstrictor). Based on these findings, we hypothesize that the upregulation of ACE2/Ang 1-7/Mas signaling could offer remarkable therapeutic benefits to COVID-19 patients with severe underlying medical conditions, which may potentially reduce morbidity and mortality among infected individuals.

## ACE2 as a SARS-coronavirus Receptor

**Figure-4:**
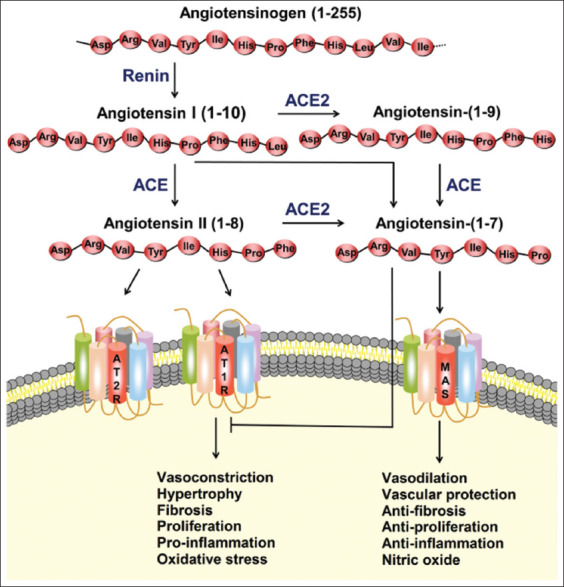
The structure of SARS-CoV-2 with different cell surface membrane proteins.

**Figure-5:**
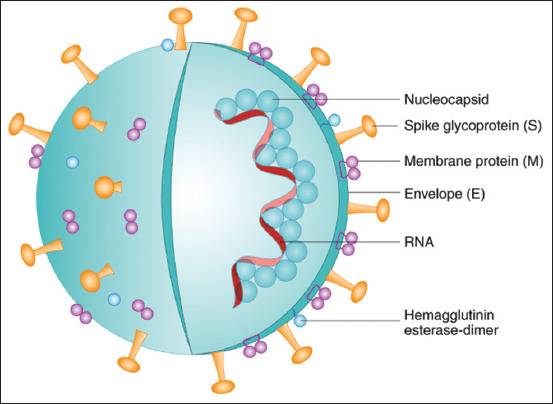
The structure of SARS-CoV-2 with different cell surface membrane proteins and the attachment of spike protein of SARS-CoV-2 with the receptor angiotensin-converting enzyme.

The current COVID-19 pandemic has drawn much attention in the past few months. Interestingly, in 2003, its predecessor, SARS-CoV, was reported to exhibit a predilection of ACE2 in *in vitro* and *in vivo* studies [26] and the successful binding of the virus to ACE2 for subsequent host cell infection. SARS-CoV in the host cell caused the downregulation of ACE2 and an increase in the local production of Ang II [27] (Figures-4 and 5). Thus far, only two coronaviruses, SARS-CoV and HCoV-NL63, require ACE2 for successful transmission [28]. The spike (S) protein of SARS-CoV-2 plays a significant key role in the receptor recognition and cell membrane fusion process. It is composed of two subunits, namely, S1 and S2. The S1 subunit contains a receptor-binding domain that recognizes and binds to the host receptor ACE2, while the S2 subunit mediates the viral cell membrane fusion into the host cell. The total length of SARS-CoV-2 is 1273 amino acids and consists of a signal peptide (amino acids 1-13) which is located at the N-terminus, whereas the S1 subunit and the S2 subunit contain 14-685 and 686-1273 amino acid residues, respectively.

## Tissue Distribution of ACE2 in Health and Disease

The ACE2 enriched organs, such as the kidneys, heart, lungs, and testis, are highly susceptible to infections, while the liver, intestine**,** and other organs containing less abundance of ACE2 are less susceptible.

## ACE2 and the Lungs

The lungs are rich in ACE2 and are highly susceptible to SARS-CoV-2 infection. Most infected patients develop pneumonia, as revealed by computerized tomography scan [29]. In fact, the lungs account for more than three quarters of ACE2 in the mammalian tissues [30]. Growing evidence has shown that in addition to respiratory distress, the derangement of the physiological function of the heart and/or kidneys often complicates disease management [31]. Moreover, the broad distribution of SARS-CoV-2 in humans is highly consistent with the pattern of ACE2 distribution [32]. Therefore, lung injury could negate pulmonary ACE2 and disrupts the Ang (1-7) ratio in favor of Ang II with the consequent exacerbation of the pathological process associated with edema in the lungs [33].

## ACE2 and Disease Conditions

### ACE2, heart failure, and coronary heart disease

**Figure-6:**
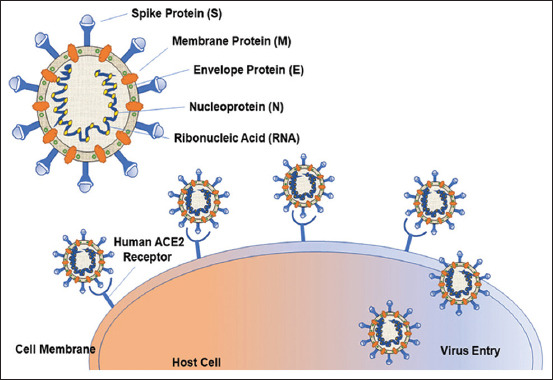
Cardiovascular involvement in coronavirus disease-19, key manifestations and hypothetical mechanisms. SARS-CoV-2 anchors on transmembrane angiotensin-converting enzyme 2 to enter the host cells leading to inflammation and organ failure.

Apart from the high mortality arising from severe respiratory distress associated with the COVID-19 pandemic, comorbidities from heart and brain dysfunctions have been reported [34]. The heart has been found to be enriched with ACE2 proteins [35], suggesting that the cardiac muscles tend to be highly susceptible to SARS-CoV-2 infection, while a preexisting heart disease before infection may predispose the affected COVID-19 patients to poor disease outcome [36]. Moreover, the physiological function of the lungs is severely compromised in COVID-19 patients and is worsened by preexisting cardiac complications [37]. Hence, the loss of ACE2 function is probably associated with cardiac dysfunctions in hospitalized patients [38]. Endothelial dysfunction, heart failure, arrhythmia, and immune system dysregulation as hypothetical pathological mechanisms through ACE2 receptor modulation in COVID-19 patients have been reported [39] (Figure-6).

## ACE2 and Hypertension

RAS dysregulation has been reported as the primary causal factor of the pathophysiology, pathogenesis, and poor prognosis of hypertension [40]. ACE2 has been shown to decrease the intravascular pressure under an experimental hypertensive condition [10]. Therefore, ACE2 could offer protection to the lungs and heart against injuries, indicating an obliteration of ACE2 protection in COVID-19 patients, along with the concomitant development of cardiovascular complications and the loss of renal function, especially in hospitalized patients [41]. The loss of ACE2 expression has been found to increase significantly in hypertension, diabetes, nephrectomy, and the lungs of patients with idiopathic pulmonary hypertension [42]. This shows that COVID-19 patients are more predisposed to renal complications and fibrosis due to the loss of ACE2 function [43]. Therefore, a treatment regimen that will enhance the expression of renal ACE2 might contribute significantly to non-fatal disease outcomes in COVID-19 hospitalized cases. Previously, a comparative study by Yamazato *et al*. [44] found that lentiviral vector-containing ACE2 treatment attenuated monocrotaline (MCT)-induced pulmonary hypertension with a reduction in pro-inflammatory cytokines in MCT-exposed mice. The finding was corroborated by an earlier finding, suggesting the positive modulatory role of ACE2 in patients with lung fibrosis [45]. To confirm this finding, Shenoy *et a*l. [46] reported the therapeutic efficacy of ACE2 in a rat model of pulmonary hypertension.

Apart from the regular respiratory symptoms associated with COVID-19, other pathologies affecting the heart, kidneys, and nervous system as well as metabolic disorders, such as diabetes, have also been reported, suggesting the involvement of the endothelium, thus as the target organ of coronavirus [47]. Recent meta-analyses have shown the effectiveness of RAS inhibitors in infected subjects, thereby reducing the level of hospitalization of patients with underlying chronic disease conditions, such as hypertension [48]. Overall, inhibiting the overexpression of ACE2 in hypertensive COVID-19 patients may contribute remarkably to the reduction in the incidence of hospitalization, morbidity, and overall mortality in the current COVID-19 pandemic [49].

## ACE2 and Chronic Kidney Disease (CKD)

In the kidneys, the ACE2 levels were high in the proximal tubular epithelial cells and endothelial cells of the renal tissue, in contrast with the glomerular cells that had low levels [50]. However, it was observed that the kidneys in healthy and disease states did not exhibit a dichotomy in the ACE2 expression in human subjects [51]. In contrast, ACE2 and ACE in rat kidneys have been found to be localized only in proximal tubules [52]. Furthermore, metabolic disorders and kidney disease have been associated with diminished ACE2 occurrence [53]. Low ACE2 vascular expression has been shown to aggravate vascular and renal diseases in recovering patients [54].

Recent studies have demonstrated that kidney impairment is commonly associated with COVID [55]. Therefore, the monitoring of sodium intake is recommended during severe COVID-19 infections, especially in patients with preexisting comorbidities [56]. Of note, end-stage kidney disease was observed in a COVID-19-infected septuagenarian [57]. Similarly, ~10% of COVID-19-infected patients could develop acute kidney injury (AKI) with poor prognosis and grave sequelae. It has been reported that COVID-19 patients with AKI tend to have a poor prognosis, accompanied by proteinuria and/or microhematuria due to the integration of the COVID-19 virus into the host cells [58]. Fulminating AKI in young patients with COVID infection has been reported [55]. The light and electron microscopic tissue examination of COVID-19 hospitalized patients revealed injuries to kidney tubules, necrosis, and the appearance of coronavirus-like particles in renal tubular epithelial cells, thus necessitating renal function monitoring in COVID-19 patients [59].

## ACE2 and Atherosclerosis

Hypertension, renal disease, and pulmonary injury are common comorbidities in COVID-19 infection. However, evidence has shown the involvement of the RAS in modifying the pathophysiology of atherosclerotic plaque deposition on vascular wall [60]. In some reports, Ang II has been reported to directly and indirectly stimulate the pathogenic processes associated with atherosclerotic plaque deposition, and ACE2 could be used as a molecular target for modifying disease outcomes in atherosclerotic patients [61,62]. In another study, ACE2 overexpression was shown to attenuate atherosclerotic plaque deposition in rabbits [63]. Likewise, the anti-atherosclerotic effect of ACE2 was demonstrated in ACE2 genetically deficient mice, probably due to reduced Ang II formation [64]. Moreover, COVID-19 infection has been shown to predispose patients to thrombotic disease, probably due to the aggravation of pro-inflammatory mechanisms, known as cytokine storm [65]. Therefore, the anti-inflammatory properties of ACE2 could be used to attenuate arteriosclerotic plaque formation in COVID-19 patients.

## Potential Therapeutic Target of ACE2

The current COVID-19 pandemic has brought economic hardship, created an unprecedented global burden, and affected the economies, educational sector, tourism, industry, and family life of people in developed, developing, and underdeveloped nations of the world. Since most organs, such as the lungs, hearts, and kidneys, compromised in COVID-19 patients are also enriched with ACE2, blocking the overexpression of ACE2 in these organs could serve as a novel therapeutic target in effectively treating COVID-19. Furthermore, urgent research is needed to identify potential drugs that could inhibit ACE2 and mitigate lung injury in COVID-19 infections. Hence, rigorous and intensive studies in blocking the expression of ACE2 should be performed, which might help eliminate, alleviate, or reverse the overall incidence of morbidity of COVID-19 patients, especially those with severe underlying medical conditions.

Recent advances in therapeutic strategies in the treatment and management of COVID-19 included the use of BIO101 (20-hydroxyecdysone, a Mas receptor activator) as a new treatment option for managing patients with SARS-CoV-2 infection at the severe stage [66]. BIO101, a 97% pharmaceutical grade 20-hydroxyecdysone, has demonstrated anti-inflammatory, antithrombotic, and antifibrotic properties by enhancing respiratory function and ultimately promoting survival in COVID-19 patients. Recently, a humanized decoy antibody (ACE2-Fc fusion protein) was designed to specifically abrogate virus replication by blocking the entry of the SARS-CoV-2 spike expressing pseudotyped virus into both ACE2 expressing lung cells and lung organoids [67]. Other intervention strategies against the new coronavirus have been reported for the development of therapies and vaccines against the new coronavirus variants [68-71]. Some potential drug candidates acting against the SARS-CoV-2 have also been reported (Table-1) [72]. These drugs have a novel capacity to reduce COVID-19 morbidity and mortality.

**Table-1:** Some potential drug candidates acting against the SARS-CoV-2 [72]

S. No.	Drug candidates	Mechanism of action
1	Remdesivir	Nucleotide analog; Broad spectrum: Many viral infections, inhibits viral RNA synthesis
2	siRNA	Short chains of dsRNA that interferes with the expression of SARS-CoV proteins
3	Camostat Mesylate	TMPRSS2 inhibitor that blocks the transmembrane protease, serine 2 (TMPRSS2) entry pathway
4	GRL0617	Inhibits PLpro activity
5	Lj001 and JL103	Induces accessory proteins membrane damage
6	Chloroquine	An antimalarial that sequesters protons in lysosomes to increase the intracellular pH
7	Lopinavir	Inhibits PLpro (3CLpro) activity
8	Pj34	Impairs viral replication

SARS-CoV=Severe acute respiratory syndrome coronavirus, PLpro=Papain-like protease

## Conclusion

Further in-depth research on the potential drugs that can serve as activators of ACE2 is needed. Recent epidemiological studies have shown that severe respiratory distress syndrome and lung injury are common symptoms of COVID-19 infection. Interestingly, since the lungs are enriched with ACE2, more research on ACE2 activation is urgently needed to improve the treatment strategies for COVID-19. Previous evidence has shown that ACE2 offered protection against lung injury and pulmonary hypertension. Furthermore, we believe that the beneficial properties of the novel ACE2 should be investigated to effectively contain COVID-19.

## Authors’ Contributions

AAO: Conceptualized the review. AAO, YGA, TOA, ITG, ADAA, AOA, OAA, TOO, OOF, TOO, ET, BSO, FOH, IOO,OEO, ABS, AAA, SMN, LJM, PNK, OOO, and MAY: Drafted, proof-read, and edited the manuscript. AAO and TOA: Performed language and plagiarism checks. All authors read and approved the final manuscript.
